# Putting the Identification of Dyslexia into a Multi-Level Perspective

**DOI:** 10.3390/brainsci10100661

**Published:** 2020-09-23

**Authors:** Pierluigi Zoccolotti

**Affiliations:** 1 Department of Psychology, Sapienza University of Rome, 00185 Rome, Italy; pierluigi.zoccolotti@uniroma1.it; 2 Neuropsychology Unit, IRCCS Fondazione Santa Lucia, 00179 Rome, Italy

There is continuing debate concerning the definition and diagnosis of dyslexia. This is partly related to the very large body of research in this area, which ranges from cognitive to biological aspects, and partly to the clinical relevance of this disorder, which has widespread implications for everyday life activities. Indeed, children with reading difficulties typically have significant problems and delays in school as well as personal problems, which typically include a failed sense of self-efficacy (e.g., [1]). In a substantial proportion of cases, additional adaptation problems emerge; these include anxiety as well as depressive symptoms (e.g., [2]).

In spite of the large body of research on dyslexia, considerable controversy still remains, even concerning the basic criteria that define the disorder. In the so-called dyslexia debate, Elliott and Grigorenko [3] raised the question of whether dyslexia exists as a separate disorder or is merely a label for an imprecisely defined clinical profile (for a historical perspective on this question, see [4]). In this journal, Protopapas and Parrila [5] questioned the use of the “neurodevelopmental disorder” umbrella to frame dyslexia. This proposal has spurred a hot debate among other researchers who are convinced of the usefulness of considering dyslexia as a neurodevelopmental disorder [6,7].

A critical theoretical perspective for framing developmental disorders was advanced in a seminal work by Morton and Frith in 1995 [8]. They proposed that developmental disorders should be viewed at a variety of levels of analysis, including the behavioral, cognitive and biological level. A number of implications follow from this proposal. In particular, using dyslexia and autism as working examples, the authors emphasized that developmental deficits should always be considered in this more general perspective even when information on some of the levels (e.g., the biological level) is missing. Note that research should be viewed by keeping the analysis of different levels separate unless interaction between levels is the specific aim of the research. For example, consider the great advancement of our knowledge on the neuroimaging of reading and reading disorders in the last 20 years (e.g., [9]). This provides groundbreaking information on what Morton and Frith [8] broadly called the biological level. However, this information does not directly define a link between brain activations and defective (and intact) cognitive processing in dyslexia unless specific links between cerebral activations and cognitive processes are investigated. This is even more the case of the initial studies that examined putative neuro-morphological differences between groups with seemingly different degrees of reading proficiency (e.g., [10]). These studies are interesting and promising but should be viewed within their level of analysis. In fact, direct links able to explain behaviorally defined symptoms (such as dyslexia) in a causative manner are still largely missing.

This seems to be the critical point raised in the paper by Protopapas and Parrila [5]. Namely, the fact that different levels of analysis are important for advancing our understanding of reading and reading difficulties is not under discussion; rather, the authors argue against the unjustified supposition that the developmental trajectories of the brain that lead to behavioral reading difficulties correspond to “failed” neural development and against the idea that one can identify a neural fault (or more than one) lying behind these trajectories. Rather, the neural states that correspond to developmental dyslexia (or the neurodevelopmental trajectories leading to them) could merely indicate benign neurodevelopmental inter-individual diversity. Indeed, the paper by Protopapas and Parrila [5] provides an analytical description of this type of research and underscores that there is still no convincing evidence that dyslexia is caused by a specific identifiable neural defect. Indeed, individual brains are differently settled, and data show that good readers’ brain activities in reading tasks only partially overlap. Thus, we are far from a unique “normative” neural developmental trajectory for reading, which might be used as a reference for defining “abnormal” development. Clearly, poor reading skill should be associated with a specific neural pattern; however, this neural pattern may vary across individuals. Certainly, we cannot rule out that a very specific biological defect will be discovered in the future; but should we call “neurodevelopmental disorder” a difficulty in which the biological and neural signature has yet to be identified? Of course, this is open to discussion and on various occasions, Fraga González, Karipidis and Tijms [6,7] have expressed their opposing view in this journal. To sum up, what seems clear is that the “neurodevelopmental disorder” label should not be assigned without explicitly stating why the definition is being given and its implications for clinical practice.

As stated above, reference to a multi-level perspective has been an important reference for framing research on developmental disorders. For example, for Pennington [11] and his research group, distinguishing different levels of analysis was crucial for establishing a foundation for the study of the partial association between developmental disorders (comorbidity), which is a key phenomenon, particularly in the case of learning disabilities. In his multiple deficit model, Pennington [11] distinguishes between four levels of analysis: etiology, brain mechanisms, cognition and behavior. Pennington [11] notes that much interaction occurs both within each of the levels of the model and across levels. Co-morbidity is seen as the partial overlap which characterizes several “complex behavioral disorders” (particularly dyslexia and deficits such as language impairment and ADHD [12]). In this model, comorbidity, seen at the behavioral level, might be associated with “*shared etiological and cognitive risk factors*”. 

Emphasizing the “behavioral” nature of the clinical description of syndromes (as they appear in international manuals such as the DSM or the ICD) has important implications for our clinical management of reading difficulties. In particular, it seems important to keep in mind that definitions of dyslexia and other developmental disorders (including autism, ADHD, etc.) are rooted in a behavioral analysis. Note that although this might seem obvious, at least in the case of dyslexia reference to a multilevel perspective [8,11] reminds us that the diagnostic definition of these disorders does not include a comprehensive description of the underlying cognitive processes (or neural/biological markers). In particular, I believe that the lack of a cognitive description might be somewhat less obvious as some of the tests used to investigate behavioral and cognitive levels are superficially similar. In fact, the diagnosis of dyslexia is based on reading tasks and to a large extent the cognitive analysis of the disorder is also based on reading tasks. 

However, the two levels of analysis, i.e., behavioral and cognitive, must be clearly distinguished. In the case of behavioral analysis, emphasis is on the quantitative evaluation and functional significance of the reading deficit (as well as on the exclusionary criteria such as sufficient educational opportunities and a lack of sensory or general intellectual problems). Is the deficit sufficiently severe to warrant a clinical diagnosis? Does it have a significant impact on the child’s efficiency in carrying out school work? Thus, here, the dimensions used refer to the severity and relevance of the reading difficulties. Clinical reading tests often use global, molar measures, such as the overall accuracy in reading lists of words or passages or global reading fluency. International diagnostic manuals clearly emphasize the importance of comparing performances in these types of tests with established normative values based on age and schooling parameters. In this perspective, dyslexia is seen as a complex behavioral disorder, characterized by poor performance on standardized reading tests and significant impairment of daily life (particular schooling) activities (see [13] for an in-depth recent analysis on this point).

The behavioral definition of dyslexia is clearly under-specified in cognitive terms. Although we know that a child is impaired in real-life reading activities, we still do not know which cognitive processes are associated with this difficulty. We also know that defective underlying cognitive processing may vary appreciably across individuals. The analysis of the cognitive processes underlying a reading deficit may provide important clues for interpreting the disorder and allow planning an individually focused intervention. This process may include several reading tests but these are viewed in a different perspective from those used to diagnose the presence of a reading disorder (as described above). Analyzing reading from a cognitive perspective is typically aimed at determining which error is made in relationship to which type of stimulus [14]. For example, the focus may be on studying how children read irregular words and whether or not they make regularization or other types of errors. Similarly, the analysis may focus on the ability to read nonsense words (with possible lexicalization errors) or infrequent words, etc. The important point here is that using molar measures of reading (such as the total number of errors made in reading a list of words) is typically of little help in the analysis of cognitive processes. Children may behave at the same level of performance in terms of global, functional measures but show quite different underlying cognitive profiles (or, alternatively, have different global performances but similar cognitive impairments).

Thus, although they may share some superficial similarities, the behavioral and cognitive analyses of reading difficulties are intrinsically different both in the type of measures/criteria and aims. Importantly, these two levels of analysis point to independent and meaningful aspects of our understanding of the disorders. In this context, the behavioral level should not be seen as an incomplete or preparatory evaluation with respect to cognitive analysis, but rather as a separate and independent step of analysis with specific goals and relying on distinct and independent dimensions (i.e., severity and functional impact).

Possibly, the separation/independence between these two levels is clearest when they yield different outcomes. An interesting example is the case of Melanie-Jane, an 80 year-old woman with a putatively marked phonological dyslexia but no history of cerebrovascular disease [15]. Melanie-Jane presented great difficulty in reading non-words, a landmark of phonological dyslexia. Nevertheless, she reported no difficulty in real-life reading or even on different formal tests that required reading lists of words. In fact, she understandably complained to the examiner: “*I could always read perfectly well and now you give me these horrible nonsense words to read*.” Is she dyslexic or not? Clearly, she does not fulfill the behavioral landmarks for the disorder and we should not conclude that she is dyslexic. Still, a detailed cognitive process analysis clearly reveals the presence of a selective difficulty in reading non-words, a hallmark of phonological dyslexia. Note also that this may not be an exceptional case. In fact, a very similar pattern was reported in a 7 year-old girl [16].

Seeing dyslexia as a “complex disorder” at the behavioral level implies that its definition is at this level of analysis, i.e., in terms of the severity and functional consequences of the reading difficulty (i.e., academic difficulties, failed sense of self-efficacy, family problems, etc.). In this view, the identification of the disorder is not directly related to specific cognitive markers. By contrast, there has been a tendency to use cognitive tests (e.g., meta-phonological tasks) as part of the criteria for defining the disorder. I think that this approach mixes behavioral and cognitive interpretations of the disorder and could lead to potential paradoxical outcomes, such as not considering as dyslexic a child with spared performance on meta-phonological tasks in the face of severe reading difficulty. 

Much of the controversy over the nature, and indeed, the existence of dyslexia might be better understood when seen in a multi-level analysis approach, a perspective originally claimed by Frith [17]. Still, the recent debate, which appeared in *Brain Sciences* [5,6,7,18], raises questions as to whether one should accept the direct implication that all levels are causally involved in dyslexia. Along these lines, Frith [17] states that “*Dyslexia can be defined as a neuro-developmental disorder with a biological origin and behavioural signs which extend far beyond problems with written language*.” By contrast, based on Protopapas and Parrila’s [5,13,18] comments, one can propose that the behavioral level is sufficient in order to efficiently and reliably define dyslexia and adding complexities inherent to other levels of analysis (such as the “neuro-developmental disorder” label or cognitive markers beyond reading) could lead to unwanted complexities in the diagnostic procedure. This does not detract at all from the idea that a full account of the reading deficit is best reached by referring to all levels of analysis (etiology, brain mechanisms, cognition and behavior).
